# A Randomized Open-Label Study of Two Methods of Proton Pump Inhibitors Discontinuation

**DOI:** 10.7759/cureus.15022

**Published:** 2021-05-14

**Authors:** Emily Hendricks, Aman N Ajmeri, Monider M Singh, Milliejoan Mongalo, Lynne J Goebel

**Affiliations:** 1 Internal Medicine, Marshall University Joan C. Edwards School of Medicine, Huntington, USA; 2 Internal Medicine, Abrazo Community Health Network, Glendale, USA; 3 Internal Medicine, Universidad Autonoma de Guadalajara School of Medicine, Guadalajara, MEX

**Keywords:** ppi, proton pump inhibitor, discontinuation, randomized trial, gerd, gastroesophageal reflux disease

## Abstract

Background

Proton pump inhibitors (PPIs) are effective in treating gastroesophageal reflux disease (GERD). Unfortunately, they are often inappropriately prescribed and long-term use has potential adverse effects. A single best method for discontinuation of PPIs does not currently exist. The objective of this study was to determine if there is a significant difference in successfully discontinuing PPI use at 12 months between patients discontinuing abruptly or tapering first.

Methodology

We conducted a randomized trial with 38 patients diagnosed with GERD. We collected six weekly and then monthly surveys of symptoms based on the Dyspepsia Symptom Severity Index. Chart review at 12 months determined whether the patient was able to discontinue PPI.

Results

A Kaplan-Meier survival analysis at 12 months did not show a statistically significant difference between the abrupt and taper groups for discontinuation of PPI medication (p = 0.75). Cox regression analysis showed no association of alcohol use, smoking, or caffeine use with failure to discontinue PPI, but H2 blocker use was associated with a 79% reduction in risk of failure to discontinue PPI (p = 0.004). The taper group had significantly less symptoms 14, 18, 22, and 30 weeks after discontinuation.

Conclusions

Our study suggests that there is no difference in successful discontinuation of PPIs between abrupt and taper methods at 12 months; however, there are less symptoms in the taper method, and H2 blocker use is associated with success. Further study is needed with larger numbers of participants and randomization of H2 blocker use.

## Introduction

Proton pump inhibitors (PPIs) are the third highest selling drug in the United States [1]. We use them to treat gastroesophageal reflux disease (GERD), a condition with a prevalence of 18-28% in North America [2]. Current GERD treatment guidelines recommend a short duration of PPI use and suggest discontinuing PPIs or maintaining the lowest effective dose [3]. Many patients, unfortunately, take PPIs for minor symptoms and become long-term users without clear indication. In fact, Bustillos et al. reported that of approximately 600 million documented PPI and histamine blocker (H2 blocker) users, only 15.8% had documentation of an appropriate indication such as heartburn, dyspepsia, gastrointestinal ulcer, erosive esophagitis, GERD, *Helicobacter pylori*, Zollinger-Ellison syndrome, or long-term non-steroidal anti-inflammatory drug (NSAID) use [4]. Taking PPIs without clear indication or for longer periods of time than necessary is problematic because long-term use is associated with increased risk of nutritional deficiencies (vitamin B12, magnesium, iron), osteoporosis, *Clostridioides difficile* infections, pneumonia, and possibly acute and chronic kidney disease [1].

Although reducing PPI use prevents harm and decreases healthcare costs, this is not always easy to do as discontinuing PPIs can cause rebound hypersecretion of acid with worsening symptoms [5]. Some patients experience heartburn, dyspepsia, and regurgitation upon discontinuation of PPIs leading them to resume use [6]. Previous studies evaluating various discontinuation strategies showed that tapering PPIs may be a better discontinuation strategy than abrupt discontinuation [7,8]. However, the majority of trials lack randomization and long-term follow-up [7].

As no guideline exists for the best method to discontinue PPIs, we undertook a study to compare two strategies, abrupt discontinuation or taper, in a randomized trial with 12 months of follow-up. The primary outcome measure was the proportion of patients who discontinued and remained off PPIs at 12 months in both groups.

## Materials and methods

Investigators obtained Institutional Review Board (IRB) approval from Marshall University IRB (IRBNet ID# 1069706-6). We conducted a prospective, randomized, open-label trial with all participants receiving education on the potential harm of continued PPI use, therapeutic management options, randomization process, procedure for filling out and returning surveys, and risks and benefits of the study. We included participants with a clinical diagnosis of GERD made by the treating physician receiving any of the following PPI medications: omeprazole (Prilosec), esomeprazole (Nexium), pantoprazole (Protonix), rabeprazole (Aciphex), lansoprazole (Prevacid), or dexlansoprazole (Dexilant) daily for at least the previous three months. Information on how the diagnosis of GERD was made was not available. Investigators screened patients from the Internal Medicine and Geriatric outpatient clinics at an academic medical center in Huntington, West Virginia. Investigators asked all eligible patients if they would like to participate and provided them with an explanation of the study and a consent form to sign. From August 2017 until December 2017, we screened 120 patients and enrolled 38 participants. Inclusion criteria were age 18 and older, diagnosis of GERD with ICD-10 K21.0 and K21.9, and taking PPI daily for at least three months. Exclusion criteria were Barrett’s esophagus, asthma, or chronic obstructive pulmonary disease, taking NSAIDs, eosinophilic esophagitis, chronic pancreatitis, pregnancy, previous failure to be able to discontinue PPI, and inability to read and understand the consent form.

We randomized participants to either an abrupt discontinuation group or a taper group. Participants in the abrupt discontinuation group stopped PPI medication immediately. Participants in the taper group taking PPI medication twice daily decreased the dose to once daily for the first two weeks, every other day for another two weeks, and then stopped the PPI. Participants in the taper group taking PPI therapy once daily took the medication every other day for two weeks and then stopped the PPI.

The initial survey consisted of baseline characteristics. On the first visit, participants received follow-up surveys to be completed and mailed in the accompanying stamped envelopes weekly for the first six weeks, and then monthly for a total of 12 months. The follow-up surveys consisted of questions regarding acid reducing medication use and symptoms using the Dyspepsia Symptom Severity Index (DSSI) [9].

Participants could use an H2 blocker such as ranitidine 150 mg once or twice daily or famotidine 20 mg once or twice daily dosed according to their estimated glomerular filtration rate and as needed calcium carbonate tablets to ease symptoms. They were asked to record use of these medications in the follow-up surveys.

We followed participants for 12 months. We called participants who did not return surveys to prompt them to do so. Even so, compliance with return of surveys was low so we used chart review to obtain most of the 12-month follow-up data and only used the first 30 weeks of survey data for symptom analysis. We reviewed physician progress notes, phone notes, and medication lists for all patients from the time of signing consent to 12 months after entering the study for evidence of PPI medication use and H2 blocker use. Investigators plotted results on a survival curve with an event defined as a participant resuming PPI medication on chart review. We defined no event as a participant remaining off PPI medication. Censored data occurred when a patient was lost to follow-up.

We excluded five participants from data analysis. Three of these participants withdrew from the taper group, one had a stroke with complications almost immediately after entrance into the study, and the other two went back on their PPI for gastroprotection for NSAID use. We excluded two participants from the abrupt group from analysis as they never stopped their PPI.

The primary endpoint was the time to resumption of PPI medications due to reemergence of symptoms at any point in time during the 12-month follow-up period. A secondary outcome was the time to resumption of PPI medications among participants who took H2 blockers to support PPI discontinuation. Other outcomes were median symptoms between taper and abrupt group.

We compared baseline characteristics by study group using the Wilcoxon rank-sum test for continuous variables and the Chi-square test (or Fisher’s exact when appropriate) for categorical variables. We calculated primary and secondary outcomes using survival analysis plotted on a Kaplan-Meier curve and compared survival statistically using the log rank test. Cox proportional hazards models were used to assess predictors of PPI resumption for the entire cohort regardless of study group. We included age, gender, caffeine use, alcohol use, smoking, H2 blocker use, and study group assignment as univariate predictors and together in the fully adjusted regression model. All analysis was performed using Stata version 16.0 (College Station, TX).

## Results

Of the 38 participants entered in the study, we randomized 20 to the taper group and 18 to the abrupt discontinuation group. A total of five participants withdrew from the study, three from the taper group and two from the abrupt group. During the 12-month follow-up, we lost one participant from the abrupt group to follow-up after the second month. Table 1 shows the characteristics of groups from the initial survey.

**Table 1:** Baseline characteristics of study groups.

Characteristics	Taper (n = 17)	Abrupt (n = 16)	P-value
Age (median)	65	57	0.008
Women n (%)	12 (71)	7 (44)	0.17
Men n (%)	5 (29)	9 (56)	
Caffeine n (%)	13 (76)	12 (75)	1.0
Smoking n (%)	0	2 (13)	0.23
Alcohol n (%)	3 (18)	4 (25)	0.69
H2 blocker use n (%)	11 (69)	13 (76)	0.7
Caucasian n (%)	16 (94)	15 (94)	1.00
Non-Caucasian n (%)	1 (6)	1 (6)	

Participants in the abrupt group were significantly younger than those in the taper group (p = 0.008). Gender did not differ between the abrupt versus taper groups (p = 0.17). Both participants who smoked were in the abrupt group. Caffeine use, alcohol intake, and H2 blocker use were similar between the two groups. The predominately Caucasian ethnicity of study participants reflects the West Virginia population demographics which is 93.5% Caucasian [10].

**Figure 1:**
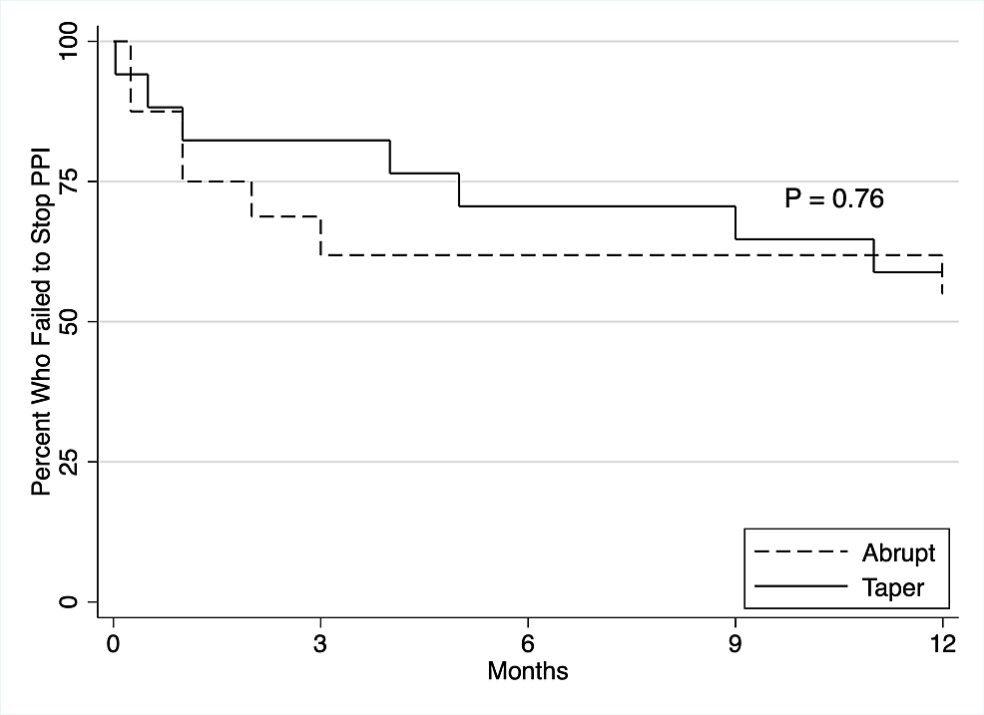
Difference in failure to stop PPI at 12 months between abrupt versus taper groups. PPI: proton pump inhibitor

A Kaplan-Meier survival analysis at 12 months (Figure 1) did not show a statistically significant difference between the taper and abrupt groups for discontinuation of PPI medications (p = 0.76), although there was a trend towards earlier failure in the abrupt group. When we combined both groups, 58% (19) were able to discontinue PPI at 12 months.

Cox regression analysis for factors associated with resuming PPI use revealed no association between age, gender, ethnicity, smoking, or alcohol use, but H2 blocker use was associated with a 79% reduction in the risk of resuming PPI (p = 0.004; hazard ratio: 0.21; 95% confidence interval [CI]: 0.073-0.62) (Table 2).

**Table 2:** Unadjusted and fully adjusted HR for restarting PPI in 33 patients on an abrupt or taper method for PPI discontinuation. *Only N = 2 observations in category outside of Caucasian which cannot be evaluated statistically with stable estimates. CI: confidence interval; HR:  hazard ratio; PPI: proton pump inhibitor

Characteristic	Unadjusted HR (95% CI)	Adjusted HR (95%CI)
Age, per year	1.02 (0.98–1.07)	1.03 (0.98–1.08)
Gender	0.48 (0.14–1.52)	0.24 (0.05–1.04)
Race*	-	-
Smoking	0.90 (0.12–6.93)	1.94 (0.16–23.3)
Alcohol use	0.52 (0.12–2.33)	0.49 (0.09–2.70)
Caffeine use	0.74 (0.23–2.38)	2.28 (0.42–12.2)
H2 blocker	0.21 (0.07–0.62)	0.18 (0.05–0.66)
Taper vs. abrupt	0.84 (0.30–2.43)	0.54 (0.13–2.23)

**Figure 2:**
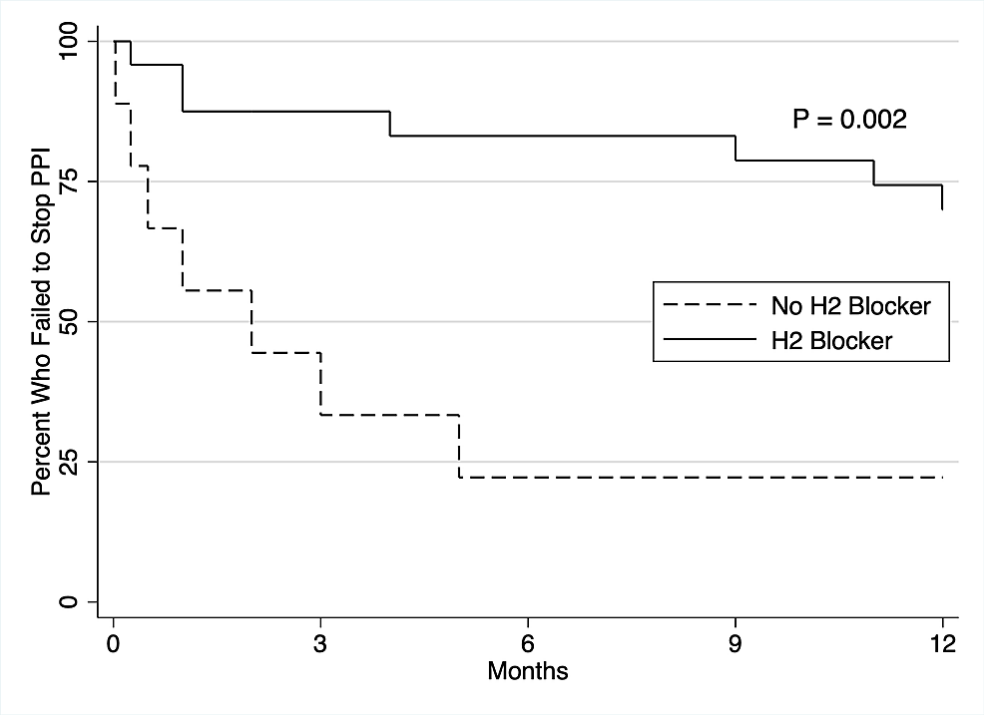
Difference in failure to stop PPI among H2 blocker users versus H2 blocker non-users. PPI: proton pump inhibitor

The participants who were not taking an H2 blocker failed faster than those using an H2 blocker (p = 0.002) (Figure 2). A total of 15 out of 18 (83%) participants who were successful at discontinuing PPI were taking H2 blockers compared to 7/15 (47%) who were not successful.

**Figure 3:**
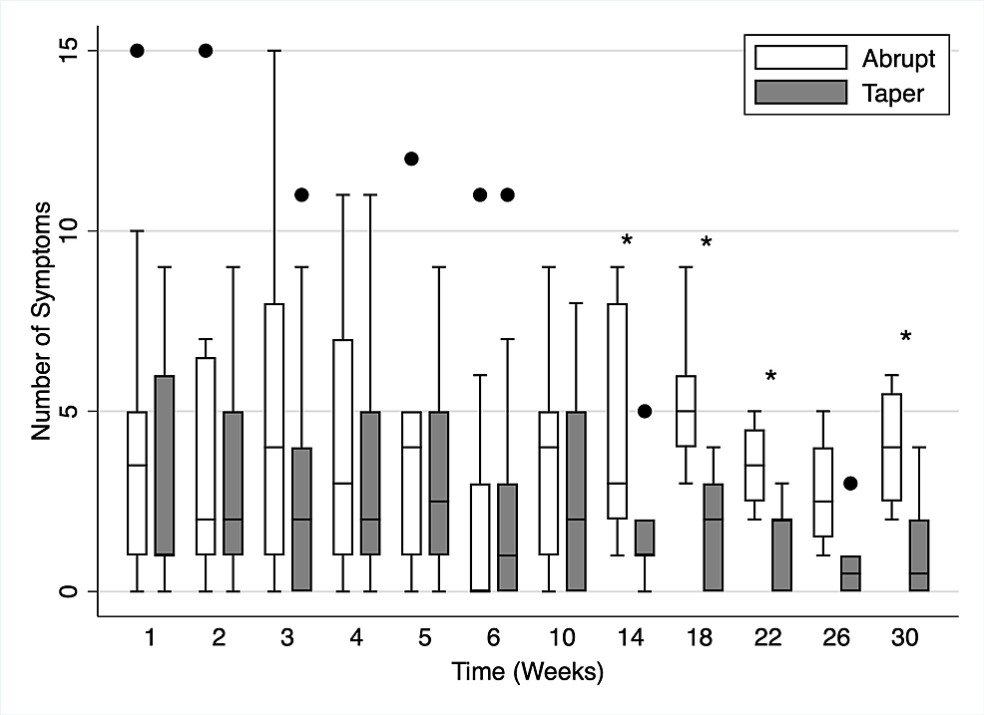
Symptoms in abrupt versus taper groups by week after discontinuation of PPI. Solid dots represent outliers. * Indicates p < 0.05. PPI: proton pump inhibitor

Symptoms based on the DSSI varied between the abrupt versus taper groups (Figure 3). The difference in the first 10 weeks was not significant. However, week 14 (p = 0.02), week 18 (p = 0.01), week 22 (p = 0.05), and week 30 (p = 0.05) showed a significant increase in symptoms occurring in the abrupt group.

## Discussion

We found no significant difference in abrupt versus taper strategy but a large effect of H2 blocker use on successful discontinuation of PPI at 12 months. Regardless of which method was used, over half of our patients were able to discontinue PPIs. In 2014, Haastrup et al. reviewed six clinical trials of de-prescribing using different methods and noted 14-64% discontinuation rates [7]. Only one of the randomized controlled studies in their review, by Bjornsson et al., was similar to our study with the exception that they used a three-week taper [11]. They also found no significant difference in discontinuation method with 22% in the abrupt versus 31% in the taper group being able to stay off PPI at one year.

Our abrupt group initially showed a more rapid return to PPI use and had significantly more symptoms than the taper group beginning at week 14 after discontinuation of PPI. This may be due to PPI-induced hypergastrinemia with gastric hyperplasia/metaplasia. Discontinuation of PPIs causes rebound acid hypersecretion (RAHS) with gastric acid levels rising above pre-treatment levels and an increase in acid-related symptoms, including dyspepsia, heartburn, and acidic regurgitation [12,13]. RAHS occurs within 14 days of discontinuation of PPI and the duration it lasts typically corresponds to the duration of PPI use [12]. Some investigators recommend every other day PPI use as it is associated with lower serum gastrin levels [14]. Moreover, it has been suggested that a longer taper may be needed for patients with higher gastrin levels [15]. There may be some people who are more sensitive to the effect of increased symptoms on stopping PPIs as only 44% of previously asymptomatic patients given PPI for four weeks developed high gastrin levels and symptoms on discontinuation [16]. Even so, almost half of our patients were unable to stop PPI due to symptom recurrence and some resumed PPI use as late as 11 months. There may be other factors that affect patients’ ability to stop their PPI. For example, all our patients used PPIs for at least three months as a criterion for entering the study, but we did not look at duration of PPI use as a factor for symptom recurrence.

In our patient population, H2 blocker use was associated with a 79% reduction in failure to discontinue PPI. We allowed use of H2 blockers and antacids as needed in both of our groups. Inadomi et al. found a step-down method of stopping PPI to be 58% successful at stopping PPI use at one year, a rate similar to our study; however, 34% still needed H2 blockers at the end of one year and only 15% were able to stay off PPI without any other medication [17]. Many of our patients asked for H2 blockers to alleviate their symptoms, and H2 blocker users showed a significant benefit for one-year success at discontinuation of PPI. Unfortunately, because we used a clinical diagnosis of GERD, it is possible that patients in our study had other diagnoses, and some may have had success if initially started on an H2 blocker. Future studies should include randomization to H2 blocker use to confirm our findings and use endoscopy to confirm the diagnosis.

There are some limitations to this study. The sample size was small and we were underpowered to detect even a moderate difference between our study groups. Patients were from one academic medical center in a city with a predominantly Caucasian population and results may not be the same for other populations. Our diagnosis of GERD was a clinical one, not substantiated by endoscopy, and people with clinical GERD may be a heterogeneous group with varied pathology. In addition, limited participant completion of surveys after six months gave us little data on symptoms for the remainder of the year.

## Conclusions

We found no significant difference in successful discontinuation of PPI medication at 12 months using abrupt or taper methods, although participants in the abrupt discontinuation group showed a trend towards earlier relapse and significantly more symptoms occurring up to six months after stopping their PPI. H2 blocker use may help with successful long-term discontinuation of PPIs. Further studies are needed with larger number of participants in a more diverse population and with randomization of H2 blocker use to confirm our results.
